# Cardiovascular disease among bariatric surgery candidates: coronary artery screening and the impact of metabolic syndrome

**DOI:** 10.1186/s13098-024-01425-6

**Published:** 2024-07-29

**Authors:** Yuntao Nie, Haoyu Zong, Zhengqi Li, Pengpeng Wang, Nianrong Zhang, Biao Zhou, Zhe Wang, Lei Zhang, Siqi Wang, Yishan Huang, Ziru Tian, Sai Chou, Xingfei Zhao, Baoyin Liu, Hua Meng

**Affiliations:** 1 Department of General Surgery & Obesity and Metabolic Disease Center, China-Japan Friendship Hospital, 2 East Yinghuayuan Street, Chaoyang District, Beijing, 100029 China; 2 Eighth Clinical School, Capital Medical University, Beijing, 100069 China; 3 Department of Oncology, Sinopharm Tongmei General Hospital, Shanxi, China; 4 School of Basic Medical Sciences, Capital Medical University, Beijing, 100069 China; 5 Department of General Surgery, Beijing Fuxing Hospital, Beijing, 100038 China

**Keywords:** Cardiovascular disease, Bariatric surgery, Obesity, Metabolic syndrome, Risk factors

## Abstract

**Background:**

Obesity is known as a risk factor for cardiovascular disease (CVD). However, there is an absence of preoperative cardiac risk assessment in bariatric surgery candidates and the incidence of CVD among these high-risk patients is still unknown.

**Methods:**

A consecutive series of bariatric surgery candidates at two Chinese tertiary hospitals received coronary CT angiography or coronary angiography from 2017 to 2023. Patients were categorized as metabolically unhealthy obesity (MUO) and metabolically healthy obesity (MHO) based on the presence or absence of MetS. CVD was diagnosed based on the maximum intraluminal stenosis > 1% in any of the segments of the major epicardial coronary arteries. Obstructive CVD was defined as coronary stenosis ≥ 50%. Binary multivariable logistic regression was performed to analyze the association between CVD and metabolic status. The number of principal MetS components was categorized into zero (without glycemic, lipid, and BP components), one (with one of the components), two (with any two components), and three (with all components) to explore their association with CVD.

**Results:**

A total of 1446 patients were included in the study. The incidence of CVD and obstructive CVD were 31.7% and 9.6%. Compared with MHO patients, MUO patients had a significantly higher incidence of mild (13.7% vs. 6.1%, *P <* 0.05), moderate (7.4% vs. 0.8%, *P* < 0.05), and severe CVD (3.1% vs. 0%, *P <* 0.05). Following complete adjustment, compared with zero or one component, two principal MetS components was found to be associated with a notable increase in the risk of CVD (OR 2.05, 95% CI 1.18–3.58, *P <* 0.05); three principal MetS components were observed to have a higher risk of CVD and obstructive CVD (OR 2.68, 95% CI 1.56–4.62, *P* < 0.001; OR 3.93, 95% CI 1.19–12.93, *P <* 0.05). Each increase in the number of principal MetS components correlated with a 1.47-fold (95% CI 1.20–1.81, *P* < 0.001) and 1.78-fold (95% CI 1.24–2.55, *P <* 0.05) higher risk of CVD and obstructive CVD, respectively.

**Conclusion:**

This study reported the incidence of CVD based on multicenter bariatric surgery cohorts. CVD is highly prevalent in patients with obesity, especially in MUO patients. Increased number of principal MetS components will significantly elevate the risk of CVD.

## Background

Obesity has emerged as a significant global health concern, with over one billion individuals worldwide diagnosed with obesity as of 2022 [1, 2]. In various countries and regions, obesity has been linked to increased all-cause mortality, predominantly due to cardiovascular disease (CVD), which accounts for the majority of deaths [3–5]. Metabolic syndrome (MetS), a prevalent comorbidity of obesity, comprises several cardiovascular risk factors—hyperglycemia, hyperlipidemia, hypertension, and central obesity—and significantly elevates the incidence and mortality associated with CVD [6, 7].

Bariatric surgery is well established as an effective treatment for obesity and MetS [8, 9]. According to the latest International Federation for the Surgery of Obesity and Metabolic Disorders (IFSO) worldwide survey, approximately 600,000 patients underwent various bariatric procedures in 2021 [10]. Given that perioperative cardiac complications will occur in 1.0–1.4% of patients, bariatric surgery should be considered an intermediate- to high-risk procedure [11]. This emphasizes the importance of preoperative cardiac evaluation. On the one hand, subclinical CVD has been found to be highly prevalent in obese individuals. According to the findings of the Framingham study [12], more than 50% of obese individuals had concomitant subclinical CVD, while this figure reached 61% in another prospective study [13]. Subclinical CVD is a precursor to overt CVD and is associated with a 2- to 8-fold elevated risk for myocardial infarction, regardless of the degree of coronary stenosis [14, 15]. On the other hand, studies based on the Metabolic and Bariatric Surgery Accreditation and Quality Improvement Program (MBSAQIP) database have indicated that MetS prolongs operation time, increases readmission rates, and significantly heightens the incidence of short-term postoperative major adverse cardiovascular events (MACE) [16]. However, despite published the American and European guidelines, coronary evaluation before bariatric surgery remains controversial and is heavily reliant on clinical experience [17, 18].

Currently, only a few small-sample studies have explored the role of coronary artery screening tests in bariatric surgery candidates, indicating that negative coronary findings have prognostic implications for ruling out MACE in the long-term postoperative period [19–21]. To our knowledge, no study has reported on the incidence of subclinical CVD in obese individuals preparing for bariatric surgery by conducting large-scale coronary artery screenings.

Therefore, our study aimed to investigate the incidence of CVD in two bariatric surgery cohorts through routine coronary artery screening and analyze the impact of the MetS on CVD.

## Methods

### Patients and study design

**Fig. 1:**
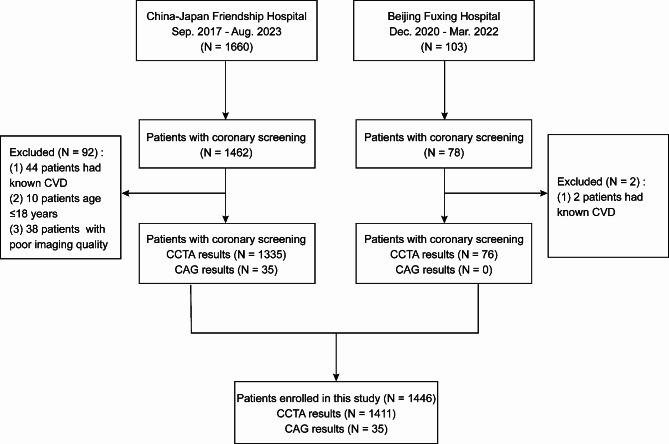
Study flow

This retrospective study was conducted on a consecutive series of patients scheduled to undergo bariatric surgery at China-Japan Friendship Hospital from September 2017 to August 2023 and Beijing Fuxing Hospital from December 2020 to March 2022. During this period, coronary artery screening, including coronary computed tomography angiography (CCTA) or coronary angiography (CAG), was routinely performed as a method of coronary evaluation in both hospitals, decided upon through multidisciplinary discussion. Patients with obesity (BMI > 27.5 kg/m^2^ according to Asian criteria) who underwent preoperative coronary artery screening were enrolled in this study. The exclusion criteria were: (1) age < 18 years; (2) patients who failed to complete coronary artery screening for any reason; (3) patients with poor imaging quality; and (4) patients with previous diagnosis of CVD or clinically manifested ischemic heart disease. The flow chart is presented in Fig. 1.

STROBE checklist was used as the reporting guide for our study. This study adhered to the Helsinki Declaration and received approval from the Institutional Review Board (IRB) of the China-Japan Friendship Hospital (2021-112-K70). Written informed consent was waived by the IRB because the study was observational and noninvasive, but verbal consent was obtained from each patient.

### Data collection

The electronic medical record system was used to extract various sociodemographic and clinical variables, including sex, age, height, weight, waist circumference, hip circumference, type 2 diabetes mellitus (T2DM), duration of T2DM, hypertension, duration of hypertension, systolic blood pressure (SBP), diastolic blood pressure (DBP), smoking history, alcohol consumption, and family history of CVD. According to the latest definition issued by European Society of Hypertension, hypertension was defined based on repeated office SBP values ≥ 140 mmHg, and/or DBP ≥ 90 mmHg, and/or previous diagnosis of hypertension, and/or preexisting use of antihypertensive medication [22]. T2DM was diagnosed according to the American Diabetes Association guidelines, including fasting plasma glucose (FPG) ≥ 7.0 mmol/L, and/or 2-h plasma glucose ≥ 11.1 mmol/L during an oral glucose tolerance test, and/or glycated hemoglobin (HbA1c) ≥ 6.5%, and/or patients with classic symptoms of hyperglycemia or a hyperglycemic crisis, random plasma glucose ≥ 11.1 mmol/L, and/or a past diagnosis of T2DM [23]. Family history of CVD was defined as the occurrence of CVD in any first-degree relatives of the individual [24].

The biochemical variables of blood samples were collected and examined within a week preoperatively, including FPG, HbA1c, triglycerides (TG), high-density lipoprotein cholesterol (HDL-C), et al. The enzymatic colorimetric method was utilized to measure the serum levels of FPG, while HbA1c was measured by high-performance liquid chromatography. TG was measured by standard enzymatic methods. The serum level of HDL-C was measured using the direct method.

### CCTA and CAG protocol

The CCTA was performed using the second-generation ostentatious dual source CT instrument of Siemens, Germany. Patients were given breath-holding training before scanning to reduce image respiratory motion artifacts and sublingual nitroglycerin three minutes before scanning to expand the coronary artery. The electrocardiogram activity of patients was monitored throughout the whole process. If the heart rate of patients exceeded 70 times per minute, about 60 mg esmolol hydrochloride injection was intravenously injected. At a speed of 5.2 ml/s, 60 ml of nonionic contrast agent of iopamidol and 50 ml of 0.9% sodium chloride injection were intravenously injected before elbow. The contrast agent tracer method was used to select the region of interest at the aortic root to monitor the CT attenuation value. When the CT attenuation value reached 100HU, it was waited for five seconds to start scanning. The scanning range was from 1 cm above aortic arch to 1 cm below cardiac diaphragm. Scanning parameters are as follows: detector collimation 1.5 × 125 × 0.5 mm. The thickness is 0.65 mm. The tube current is 350 mAs/turn. Rotation time is 0.3 s/cycle, and tube voltage is 120 kV.

The CAG was performed in patients with a supine position using the transradial cardiac catheterization procedure and screened by a digital angiography platform (Innova3100, GE Healthcare, Chicago, USA). A patient was injected with 40 ml of contrast medium of iodixanol 320 mgl/ml (Visipaque, GE Healthcare, Cork, Ireland) at 5 ml/second before coronary angiogram. The scanning parameters were: 120 − 140 kVp; 50 − 150 mAs; matrix size, 512 × 512 pixels; field of view, 16 cm; Lao projection: 23 − 46 degrees; Rao projection: 16 − 41 degrees.

### Coronary artery imaging assessment

All CCTA scans were analyzed by two experienced radiologists, each with over 10 years of expertise. Results from the CAG were collected via chart review, which included information on the location and degree of CVD severity as reported by the performing cardiologists. Angiographic analysis was performed using the AHA 17-segment model [25]. Segments were included in the analysis if their diameter was > 1.5 mm. The severity of luminal diameter stenosis was classified as none (0%), minimal (1–24%), mild (25–49%), moderate (50–69%), and severe (≥ 70%) [26]. CVD was diagnosed based on the maximum intraluminal stenosis > 1% in any of the segments of the major epicardial coronary arteries. Obstructive CVD was defined as coronary stenosis ≥ 50%, and significant obstructive CVD was defined as coronary stenosis ≥ 70%. The number of diseased vessels was classified as one, two, three, or left main (LM) coronary artery [27].

### Definition of MetS

According to the International Diabetes Federation (IDF) criteria [28], metabolic syndrome was diagnosed as a combination of three or more of the following five components: (1) central obesity (waist circumference ≥ 90 cm in men or ≥ 80 cm in women); (2) triglycerides ≥ 1.7 mmol/L and/or specific treatment for this lipid abnormality; (3) HDL-C < 1.03 mmol/L in men or < 1.29 mmol/L in women and/or specific treatment for this lipid abnormality; (4) SBP ≥ 130 mmHg or DBP ≥ 85 mmHg and/or treatment of previously diagnosed hypertension; and (5) FPG ≥ 5.6 mmol/L and/or previously diagnosed T2DM. Patients were categorized based on their metabolic status into two groups: metabolically healthy obesity (MHO; without MetS) and metabolically unhealthy obesity (MUO; with MetS). Since all patients met the central obesity component, the remaining four components were reduced to three principal components (glycemic, lipid, and blood pressure [BP] components), with reduced HDL-C and elevated triglycerides being combined into the lipid component. The number of principal components was categorized into zero (without glycemic, lipid, and BP components), one (with one of the components), two (with any two components), and three (with all components) to explore their association with CVD.

### Statistical analysis

Continuous variables were presented as means with standard deviations for normally distributed data, and medians with interquartile ranges (IQR) for non-normally distributed data, while categorical variables were reported as counts and proportions. Continuous variables were compared between groups using the Student t-test or Mann-Whitney U test. Categorical variables were compared using the Chi-square test or Fisher exact test. Binary multivariable logistic regression analysis was conducted to explore the association between the presence of CVD and the number of principal MetS components. The odds ratios (ORs) along with corresponding 95% confidence intervals (CIs), were presented as outcomes obtained from the logistic regression model. We constructed three models with adjustments for major covariates: Unadjusted Model 1; Model 2, which adjusted for sex, age, and BMI; and Model 3, which additionally adjusted for all clinical variables except those directly related to MetS components (hypertension, SBP, DBP, duration of hypertension, T2DM, duration of T2DM). Subgroup analyses were conducted to assess whether potential covariates (sex, age, BMI, waist-to-hip ratio, smoking history, alcohol consumption) modified the relationship between the number of principal MetS components and CVD. The significance level was set at *P* < 0.05 for all tests. Data analysis was performed using SPSS version 24 and R 4.1.3 software.

## Results

### Patient characteristics

Table 1 presents the patient characteristics. A total of 1446 patients with obesity were included in the study, predominantly female (67.7%), with a mean age of 37.0 ± 9.5 years and a median BMI of 37.2 (IQR, 32.8–42.0) kg/m^2^. The prevalence of hypertension and T2DM was 56.6% and 65.4%, respectively, and there was a family history of CVD in 5.3% of the patients.

**Table 1:** Patient characteristics

Characteristic	Total (*n* = 1446)	MUO (*n* = 1314)	MHO (*n* = 135)	*P* Value
Male, n (%)	467 (32.3)	443 (33.7)	24 (18.3)	< 0.001
Age (years)	37.0 ± 9.5	37.4 ± 9.6	32.7 ± 8.1	< 0.001
Height (cm)	167.8 ± 8.5	167.9 ± 8.6	166.7 ± 7.4	0.088
Weight (kg)	108.1 ± 24.2	108.4 ± 24.3	105.4 ± 23.4	0.171
BMI (kg/m^2^)	37.2 (32.8, 42.0)	37.2 (32.8, 42.1)	36.8 (33.0, 40.1)	0.417
Waist circumference (cm)	117.4 ± 15.8	117.8 ± 15.9	113.3 ± 14.9	< 0.050
Hip circumference (cm)	120.9 ± 13.9	120.7 ± 13.9	122.8 ± 13.3	0.106
Waist-hip ratio	1.0 ± 0.1	1.0 ± 0.1	0.9 ± 0.1	< 0.001
Smoking history, n (%)	318 (22.0)	298 (22.7)	20 (15.3)	0.051
Smoking index (pack-years)	10.0 (3.6, 20.0)	10.0 (3.8, 20.0)	8.8 (1.0, 22.7)	< 0.050
Alcohol consumption, n (%)	216 (14.9)	202 (15.4)	14 (10.7)	0.152
Hypertension, n (%)	819 (56.6)	798 (60.7)	21 (16.0)	< 0.001
SBP (mmHg)	138.4 ± 20.1	139.7 ± 19.9	124.7 ± 16.3	< 0.001
DBP (mmHg)	87.2 ± 14.0	88.0 ± 14.1	79.2 ± 10.7	< 0.001
Duration of hypertension (year)	1.0 (0.0, 5.0)	1.0 (0.0, 5.0)	0.0 (0.0, 1.0)	< 0.001
T2DM, n (%)	946 (65.4)	933 (71.0)	13 (9.9)	< 0.001
Duration of T2DM (year)	1.0 (0.0, 5.0)	1.0 (0.0, 5.0)	1.0 (0.1, 4.5)	< 0.001
Family history of CVD, n (%)	77 (5.3)	71 (5.4)	6 (4.6)	0.691
FPG (mmol/L)	7.3 ± 3.0	7.5 ± 3.0	5.2 ± 1.1	< 0.001
HbA1c (mmol/L)	7.0 ± 1.8	7.2 ± 1.9	5.7 ± 0.8	< 0.001
TG (mmol/L)	2.3 ± 1.9	2.5 ± 1.9	1.2 ± 0.5	< 0.001
HDL-C (mmol/L)	1.1 ± 0.3	1.1 ± 0.3	1.2 ± 0.2	< 0.001
LDL-C (mmol/L)	3.0 ± 0.7	3.0 ± 0.7	2.9 ± 0.6	0.071

Continuous variables were compared between groups using the Student t-test or Mann-Whitney U test. Categorical variables were compared using the Chi-square test or Fisher exact test

Abbreviations: BMI, body mass index; DBP, diastolic blood pressure; FPG, fasting plasma glucose; HbA1c, glycosylated hemoglobin A1c; HDL-C, high-density lipoprotein cholesterol; LDL-C, low-density lipoprotein cholesterol; MHO, metabolically unhealthy obesity; MUO, metabolically unhealthy obesity; SBP, systolic blood pressure; T2DM, type 2 diabetes mellitus; TG, triglyceride

**Fig. 2:**
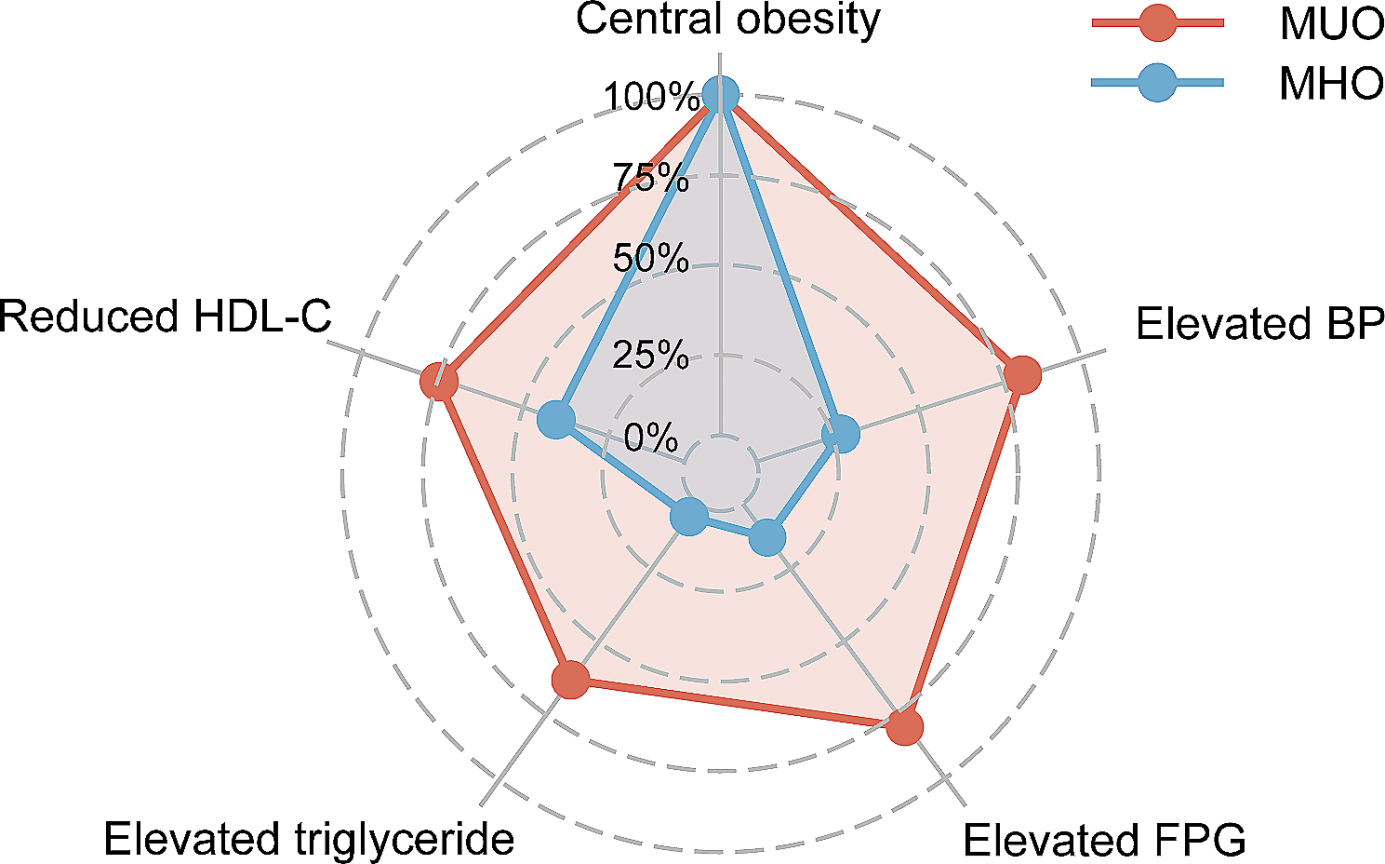
MetS components of MUO and MHO patients Abbreviations: BP, blood pressure; FPG, fasting plasma glucose; HDL-C, high-density lipoprotein cholesterol; MHO, metabolically unhealthy obesity; MUO, metabolically unhealthy obesity

**Fig. 3:**
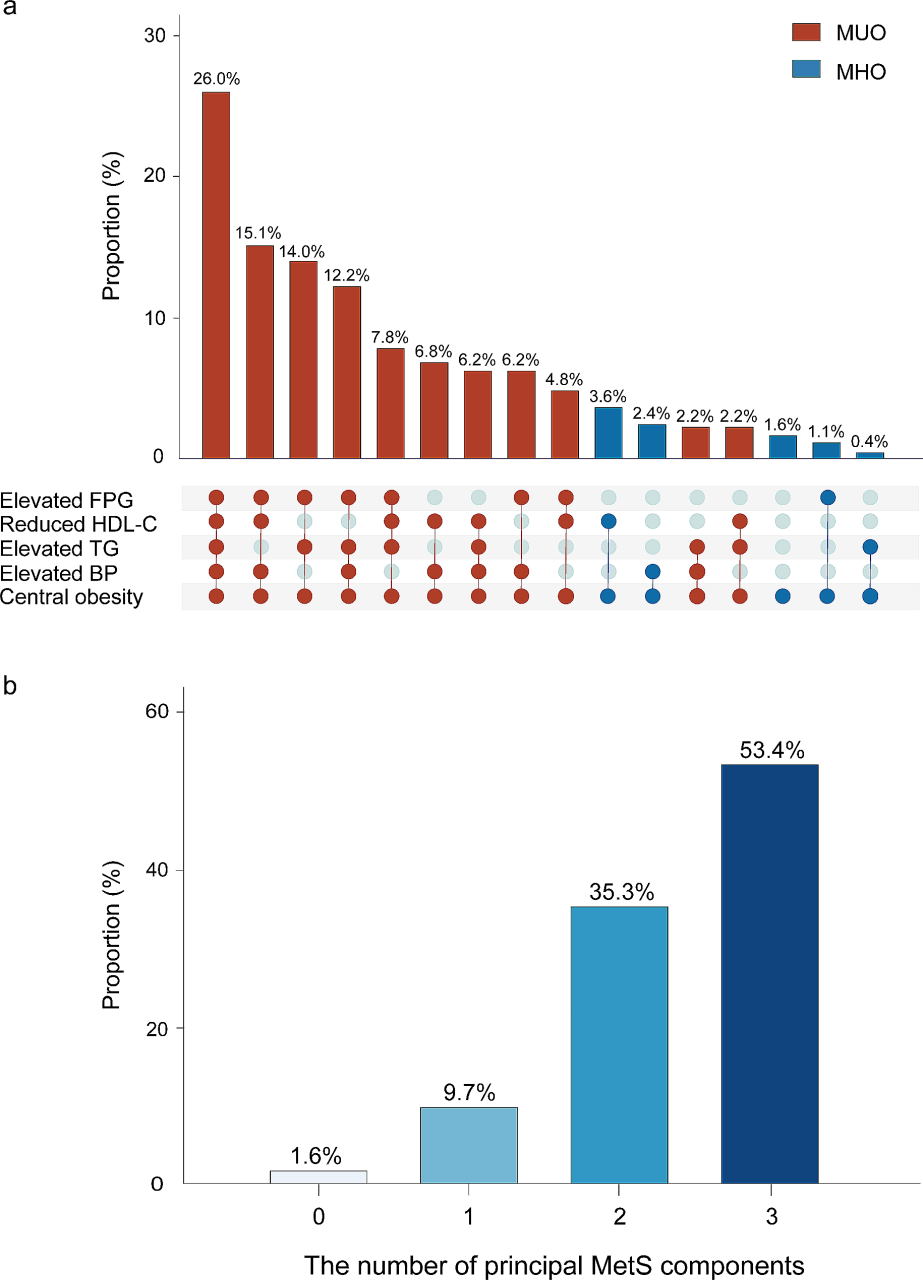
The distribution of (**a**) MetS components (**b**) principal MetS components Abbreviations: BP, blood pressure; FPG, fasting plasma glucose; HDL-C, high-density lipoprotein cholesterol; TG, triglyceride; MHO, metabolically unhealthy obesity; MUO, metabolically unhealthy obesity

Of all patients, 1315 (90.9%) were categorized as MUO, and the remaining 131 (9.1%) as MHO. MUO patients were typically male, older, and had higher waist circumferences, waist-to-hip ratios, and smoking index, along with poorer metabolic profiles including higher FPG, HbA1c, triglycerides, HDL-C, and LDL-C levels. Moreover, MUO patients were more prone to having hypertension and T2DM. Figure 2 illustrates the proportion of MetS components met by the MHO and MUO patients. All the patients had central obesity. Compared with the MHO patients, MUO patients had a higher proportion of elevated BP (82.1% vs. 26.0%, *P* < 0.001), elevated FPG (80.9% vs. 12.2%, *P* < 0.001), elevated triglycerides (63.8% vs. 4.6%, *P* < 0.001), and lowered HDL-C (75.8% vs. 39.7%, *P* < 0.001). The distribution of MetS components was shown in Fig. 3a. According to our classification, the proportion of patients with zero, one, two, and three principal MetS components was 1.6%, 9.7%, 35.3%, and 53.4%, respectively. (Fig. 3b).

### Coronary artery imaging findings

Following multidisciplinary discussions, 1411 (97.6%) patients underwent cardiac risk assessment via CCTA, while 35 (2.4%) patients underwent CAG. The coronary artery imaging findings are shown in Table 2. CVD was identified in 459 (31.7%) of the 1446 patients, with 139 (9.6%) presenting obstructive CVD and 41 (2.8%) presenting significant obstructive CVD. The incidence rates for 1-vessel, 2-vessel, and 3-vessel or LM disease were 6.6%, 2.1%, and 0.8% respectively in patients with obstructive CVD, and 2.3%, 0.5%, and 0.1% respectively in patients with significant obstructive CVD.

**Table 2:** Coronary artery imaging findings

Variable	Total population (*n* = 1446)
CVD	459 (31.7%)
Minimal (1–24%)	132 (9.1%)
Mild (25–49%)	188 (13.0%)
Moderate (50–69%)	98 (6.8%)
Severe (≥ 70%)	41 (2.8%)
Obstructive CVD	139 (9.6%)
1VD	96 (6.6%)
2VD	31 (2.1%)
3VD or LM disease	12 (0.8%)
Significant obstructive CVD	41 (2.8%)
1VD	33 (2.3%)
2VD	7 (0.5%)
3VD or LM disease	1 (0.1%)

Obstructive CVD is defined as ≥ 50% maximal diameter stenosis, significant obstructive CVD is defined as ≥ 70% maximal diameter stenosis.

Abbreviations: CVD, coronary artery stenosis; LM, left main coronary artery disease; 1VD, 1- vessel disease; 2VD, 2- vessel disease; 3VD, 3- vessel disease.

### Correlation between MetS and CVD

**Fig. 4:**
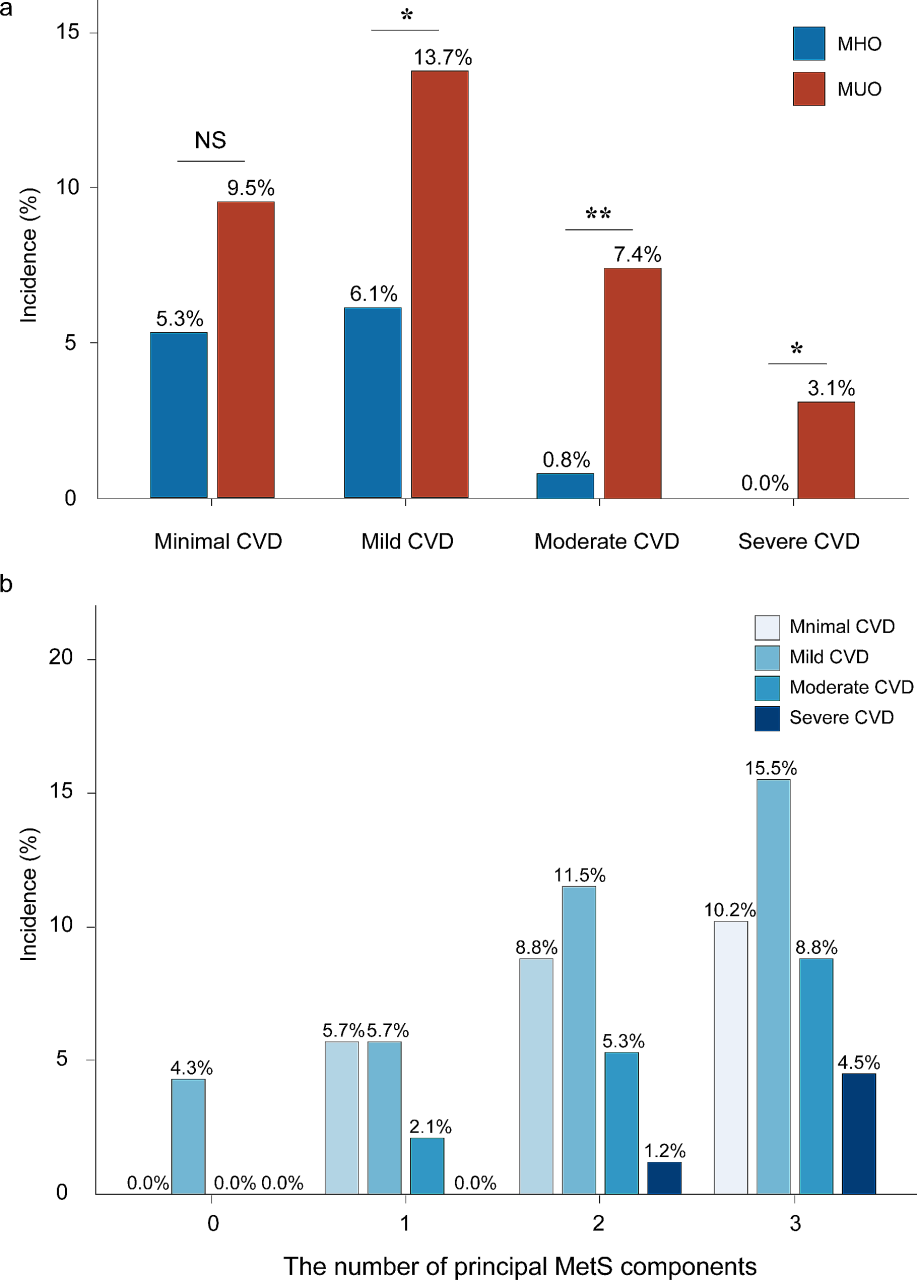
The incidence of CVD (**a**) between MUO and MHO patients (**b**) among different numbers of principal MetS components. Differences between the two groups were calculated by using Chi-square test Abbreviations: CVD, cardiovascular disease; MUO, metabolic unhealthy obesity; MHO, metabolic healthy obesity; MetS, metabolic syndrome; NS, no significant; *P < 0.05, **P < 0.01,***P < 0.001

Figure 4a displays the incidence of CVD in MUO and MHO patients. Compared with MHO patients, MUO patients had a significantly higher incidence of mild (13.7% vs. 6.1%, *P <* 0.05), moderate (7.4% vs. 0.8%, *P* < 0.05), and severe disease (3.1% vs. 0%, *P* < 0.05). Notably, 10.5% of MUO patients had obstructive CVD, significantly higher than MHO patients, only 1 (0.8%) of whom had obstructive CVD (*P* < 0.001). Figure 4b shows an increase in the occurrence of CVD of varying severity with the increase of principal MetS components. The incidences of obstructive CVD in patients with zero, one, two and three principal MetS components were 0.0%, 2.1%, 7.4% and 13.3%. Furthermore, the corresponding incidences of significant obstructive CVD were 0.0%, 0.0%, 1.2% and 4.5%, respectively.

Table 3 displays the relationship between the number of principal MetS components and CVD. In univariable logistic regression (Model 1), patients with two principal MetS components were found to be associated with a notable increase in the risk of CVD and obstructive CVD (OR 2.62, 95% CI 1.58–4.35, *P* < 0.001; OR 3.68, 95% CI 1.11–12.17, *P <* 0.05); three principal MetS components was observed to have a higher risk of CVD and obstructive CVD (OR 4.59, 95% CI 2.82–7.50, *P* < 0.001; OR 8.21, 95% CI 2.57–26.22, *P* < 0.001) compared to patients with zero or one component. After accounting for sex, age, and BMI, a similar positive correlation was observed (Model 2). In the fully adjusted model (Model 3), two principal MetS components exhibited a significantly elevated risk of CVD (OR 2.05, 95% CI 1.18–3.58, *P* < 0.05); three principal MetS components were linked to higher odds ratios for CVD and obstructive CVD (OR 2.68, 95% CI 1.56–4.62, *P* < 0.001; OR 3.93, 95% CI 1.19–12.93, *P* < 0.05). Likewise, each increase in the number of principal MetS components correlated with a 1.47-fold (95% CI 1.20–1.81, *P* < 0.001) and 1.78-fold (95% CI 1.24–2.55, *P* < 0.05) higher risk of CVD and obstructive CVD, respectively.

**Table 3:** Odds ratios (95% CIs) of CVD according to the number of principal MetS components

	The number of principal MetS components			*P* trend	Each increase in number of principal MetS components
	0 or 1	2	3		
CVD					
Model 1	1.00	2.62 (1.58–4.35)	4.59 (2.82–7.50)	< 0.001	1.95 (1.62–2.34)
Model 2	1.00	1.96 (1.15–3.34)	2.62 (1.57–4.39)	< 0.050	1.48 (1.22–1.81)
Model 3	1.00	2.05 (1.18–3.58)	2.68 (1.56–4.62)	< 0.001	1.47 (1.20–1.81)
Obstructive CVD					
Model 1	1.00	3.68 (1.11–12.17)	8.21 (2.57–26.22)	< 0.001	2.44 (1.74–3.42)
Model 2	1.00	2.36 (0.70–7.93)	4.18 (1.29–13.60)	< 0.050	1.87 (1.31–2.66)
Model 3	1.00	2.34 (0.69–7.96)	3.93 (1.19–12.93)	< 0.050	1.78 (1.24–2.55)

Odds ratio (OR) and 95% confdence interval (CI) was evaluated using binary multivariable logistic regression models

Model 1: not adjusted.

Model 2: model 1 + adjusted for gender, age and BMI

Model 3: model 2 + adjusted for hip circumference, smoking history, smoking index, alcohol consumption and Family history of CVD

Abbreviations: BMI, body mass index; CI, confidence intervals; CVD, cardiovascular disease; OR, odds ratios

### Subgroup analyses for the association between the number of principal MetS components and CVD

**Fig. 5:**
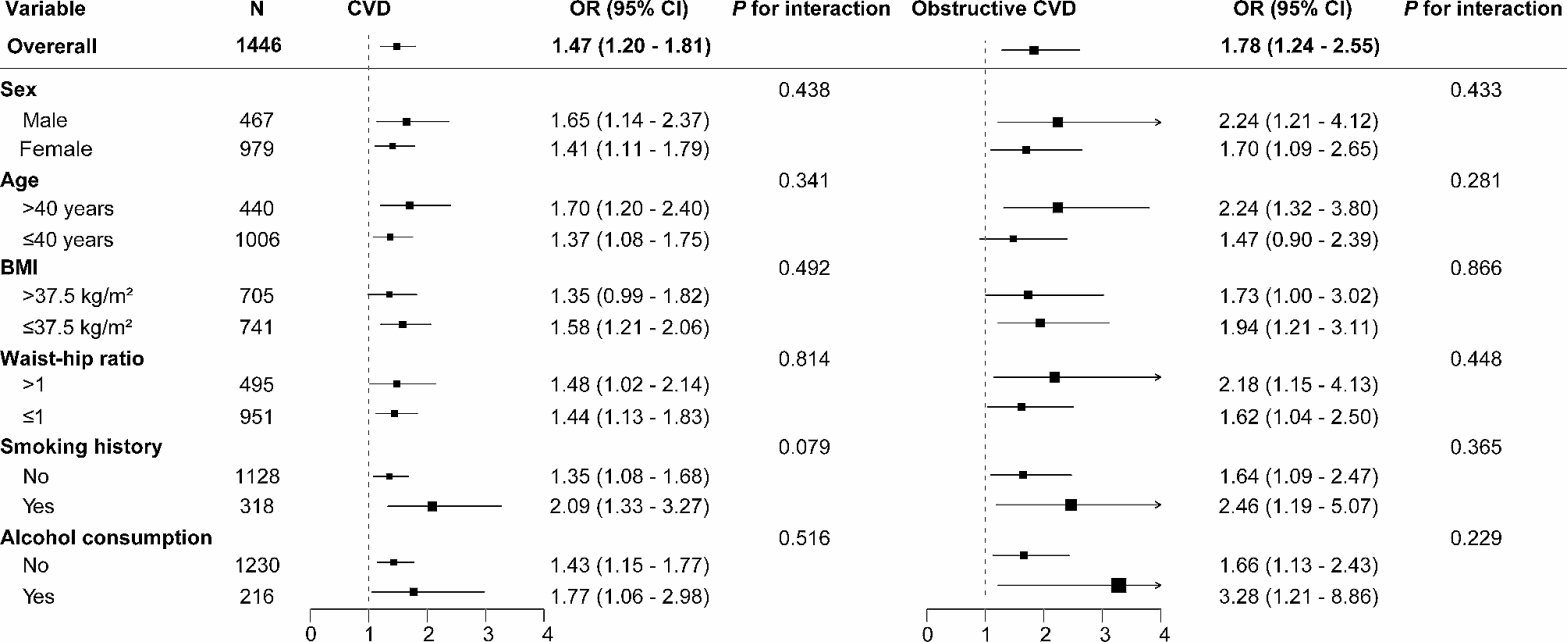
Subgroup analyses of the association between CVD and the number of principal MetS components. Adjusted for age, sex, BMI, hip circumference, smoking history, smoking index, alcohol consumption, and Family history of CVD Abbreviations: CI, confidence interval; CVD, cardiovascular disease; OR, odds ratio

The subgroup analyses indicated that principal MetS components were positively associated with CVD and obstructive CVD in most subgroups, stratified by sex, age, BMI, waist-to-hip ratio, smoking history, and alcohol consumption. No significant interactions between the number of principal MetS components and these potential CVD risk factors for interest were observed (all *P* for interaction > 0.05) (Fig. 5).

## Discussion

Despite patients with obesity undergoing bariatric surgery being vulnerable to CVD risk factors, preoperative coronary artery screening is often neglected in clinical practice [29, 30]. None of the current studies has addressed the burden of subclinical CVD in candidates for bariatric surgery, which is crucial for perioperative safety and long-term cardiovascular prognosis. Our study reported, for the first time, that the incidence of CVD exceeded 30% in two bariatric surgery cohorts through routine coronary artery screening, with the incidence of obstructive CVD approaching 10%. MetS significantly increased the occurrence and severity of CVD in obese individuals, whereas MHO patients have minimal to no risk of developing CVD. Upon reducing the MetS components to three principal components—BP, glycemia, and lipids—an increase in these components was associated with a higher risk of CVD.

Historically, insights into the natural progression of CVD have been derived from autopsy observations and patients undergoing CAG due to symptoms of myocardial ischemia [31–33]. Nonetheless, CVD that exhibits obvious symptoms is always the tip of the iceberg, while the burden of asymptomatic subclinical CVD remains uncertain [12, 34]. Data from the Veterans Affairs (VA) Clinical Assessment, Reporting, and Tracking (CART) program revealed that non-obstructive CVD, relative to no apparent CVD, correlated with notably heightened 1-year risks of myocardial infarction and all-cause mortality [15]. Another recent large-scale investigation, the Copenhagen General Population Study, demonstrated that subclinical obstructive CVD was linked to 8-fold increased risk of myocardial infarction in an asymptomatic cohort during a median follow-up of 3.5 years [14]. These results suggested that subclinical CVD still contributes to increased cardiovascular events and mortality, highlighting the importance of early screening and intervention to enhance prognosis.

Guidelines established by the American College of Cardiology (ACC) and the American Heart Association (AHA) recognize obesity as a significant modifiable cardiovascular risk factor for secondary prevention of CVD [17]. Bariatric surgery has been extensively demonstrated as one of the most effective interventions for treating obesity and related comorbidities [35–37]. Given that individuals with obesity commonly present with multiple cardiovascular risk factors, thorough coronary evaluation prior to bariatric surgery is imperative. However, the majority of existing studies have neglected preoperative coronary evaluation or solely conducted imaging screening on a select few patients identified as being at high cardiac risk through risk assessment tools, thus overlooking numerous patients with subclinical CVD [20, 38]. Lubanski et al. [13] performed CCTA on 41 obese individuals with an average age of 50.4 years and BMI > 40 kg/m^2^, revealing the presence of subclinical CVD in 61% of the study population. Tognolini et al. [21] detected coronary stenosis in 30 consecutive candidates for bariatric surgery using cardiac dual-source CT, with subclinical CVD observed in 33% of the participants. Consistent with previous research, our investigation, encompassing 1446 patients from two bariatric surgery cohorts who underwent routine coronary artery screening, revealed a prevalence of subclinical CVD at 31.7%, with 9.6% of these individuals afflicted by obstructive CVD. To the best of our knowledge, this study represents the most extensive coronary artery screening investigation conducted within bariatric surgery cohorts to date, thereby offering more precise evidence.

The high incidence of CVD in our study supports previous research findings that maintaining a healthy weight has a protective effect on the cardiovascular system. Bogers et al. [39] have shown that healthy body weight reduces the risk of CVD compared to higher BMI. At the same time, an increase in body weight is accompanied by an increase in MetS components [40]. Da Hea et al. [41] and Yoo-Bin et al. [16]. have found increased cardiovascular risk in MHO individuals compared to metabolically healthy normal weight, and a further increase in CVD risk in MUO compared to MHO, confirming that maintaining a healthy body weight can both reduce the incidence of metabolic syndrome and provide cardiovascular protection .

Among the various comorbidities of obesity, MetS stands out as a condition strongly linked to CVD, characterized by a cluster of cardiovascular risk factors [42, 43]. Chinese patients opting for bariatric surgery as a treatment often exhibit a higher prevalence of MetS, attributed to health insurance restrictions and conservative attitudes toward surgery, as evidenced by the finding that over 90% of the patients categorized as MUO in this study. In comparison to MHO patients, MUO patients have been documented to display more severe impairment of microvascular function and experience higher rates of cardiovascular morbidity and all-cause mortality [44, 45]. In line with these findings, our results suggest that MUO patients exhibit a higher prevalence of CVD than MHO patients across nearly all severity levels, indicating that MetS could serve as a potential predictor of cardiac risk in obese individuals. A recent study based on the MSBAQIP database also found that patients with MetS had a 3-fold higher risk of MACE during the perioperative period of bariatric surgery than patients without MetS, indicating that obesity combined with MetS constitutes a significant concern for coronary artery screening [46].

Interestingly, the incidence of obstructive CVD in MHO patients was below 1% in both bariatric surgery cohorts, though this result should be interpreted with caution due to the relatively low proportion of MHO in the total population. Similarly, several previous studies have shown that MHO is associated with a lower cardiovascular risk compared to MUO, yet is comparable to metabolically healthy normal weight counterparts [15, 47, 48]. A recently published study by Petersen et al. suggested that the primary distinction in cardiovascular risk between MHO and MUO patients is attributable to specific cardiometabolic characteristics of MHO patients [49]. These included altered skeletal muscle biology (decreased ceramide content and increased expression of genes involved in branched-chain amino acid catabolism and mitochondrial structure/function), altered adipose tissue biology (reduced expression of genes involved in inflammation and extracellular matrix remodeling and increased expression of genes related to lipogenesis), lower 24-hour plasma glucose, insulin, non-esterified fatty acids, and triglycerides; higher plasma adiponectin and lower plasma plasminogen activator inhibitor-1 (PAI-1) concentrations; and reduced oxidative stress. In summary, MetS diagnosed using IDF criteria can serve as a reliable indicator for coronary artery screening and accurately stratify the cardiac risk.

The number of MetS components, referred to as the MetS score in other studies, is positively associated with atherosclerosis, T2DM, carotid intima-media thickening, and CVD mortality [50, 51]. Central obesity, which is prevalent in Asian populations, is characterized by increased intra-abdominal fat and significantly elevates the risk of metabolic abnormalities [52, 53]. Candidates for bariatric surgery typically meet the criterion for central obesity; therefore, we consolidated the four additional MetS components into three principal elements—BP, glycemia, and lipid components—to investigate their association with cardiovascular risk. We determined that the number of principal MetS components is independently associated with CVD after adjusting for various clinical characteristics. For each additional principal MetS component, there was a 47% increase in CVD risk and a 78% increase in obstructive CVD risk. Having all three principal MetS components fulfilled further increases the risk of CVD and obstructive CVD. These findings indicate that an increased number of major MetS components is positively correlated with the severity of CVD.

This study carries significant clinical implications. On the one hand, this is the first large-scale coronary artery screening study based on multicenter bariatric surgery cohorts to report the prevalence of subclinical CVD, thus providing evidence-based support for preoperative cardiac evaluation. On the other hand, this study indicates that MetS can be effectively utilized for cardiac risk stratification of bariatric surgery candidates. MHO patients, being at lower cardiac risk, could potentially be exempted from preoperative coronary imaging tests. In contrast, MUO patients, especially those with a high number of principal MetS components, are advised to undergo preoperative coronary assessment to screen for potentially risky CVDs, enabling early intervention and close monitoring to reduce the incidence of MACE and CVD mortality.

There are a few limitations in this study. First, given its retrospective design, the study is inevitably influenced by selection bias. Second, the final decision on whether to perform coronary assessment using CCTA or CAG was made by a multidisciplinary team comprising anesthesiologists, bariatric surgeons, cardiologists, and endocrinologists. This inherently subjective process prevents a direct comparison of the diagnostic efficacy between the two techniques. Third, long-term cardiovascular events were not reported in this study due to insufficient follow-up time, which precluded correlating screened subclinical CVD with outcomes. Fourth, since all participants were Asian, it remains uncertain whether these findings can be generalized to Western populations.

## Conclusion

The incidence of CVD in bariatric surgery candidates was 31.7%, and the incidence of obstructive CVD was 9.6% by CCTA and CAG screening. MetS can significantly elevate CVD incidence, and the higher the number of principal MetS components, the higher the cardiovascular risk. These findings emphasize the importance of preoperative coronary artery screening in candidates for bariatric surgery, providing evidence for perioperative cardiac management in this field.

## Data Availability

The datasets generated and/or analyzed during our study are available from the corresponding author upon reasonable request.

## Abbreviations

ACC: American College of Cardiology
AHA: American Heart Association
BMI: Body mass index
CAG: Coronary angiography
CART: Clinical Assessment, Reporting, and Tracking
CCTA: Coronary CT angiography
CI: Confidence intervals
CVD: Cardiovascular disease
DBP: Diastolic blood pressure
FPG: Fasting plasma glucose
HbA1c: Glycosylated hemoglobin A1c
HDL-C: High-density lipoprotein cholesterol
IFSO: International Federation for the Surgery of Obesity and Metabolic Disorders
LDL-C: Low-density lipoprotein cholesterol
MACE: Major adverse cardiovascular events
MBSAQIP: Metabolic and Bariatric Surgery Accreditation and Quality Improvement Program
MetS: Metabolic syndrome
MHO: Metabolically healthy obesity
MUO: Metabolically unhealthy obesity
OR: Odds ratios
PAI-1: Plasminogen activator inhibitor-1
SBP: Systolic blood pressure
STROBE: STrengthening the Reporting of OBservational studies in Epidemiology
T2DM: Type 2 diabetes mellitus
TG: Triglyceride
VA: Veterans Affairs
